# Revealing the lethal effects of *Pasteurella multocida* toxin on multiple organ systems

**DOI:** 10.3389/fmicb.2024.1459124

**Published:** 2024-08-27

**Authors:** Jianlin Yuan, Jinfeng Li, Senyan Du, Yiping Wen, Yiping Wang, Yi-Fei Lang, Rui Wu, Qi-Gui Yan, Shan Zhao, Xiaobo Huang, Qin Zhao, San-Jie Cao

**Affiliations:** ^1^Research Center for Swine Diseases, College of Veterinary Medicine, Sichuan Agricultural University, Chengdu, China; ^2^Engineering Research Center of Southwest Animal Disease Prevention and Control Technology, Ministry of Education, Chengdu, China; ^3^Sichuan Science-Observation Experimental Station of Veterinary Drugs and Veterinary Diagnostic Technique, Ministry of Agriculture and Rural Affairs, Chengdu, China

**Keywords:** *Pasteurella multocida* toxin (PMT), cytotoxicity, animal lethality, organ damage, nephrotoxicity in pigs

## Abstract

*Pasteurella multocida* toxin (PMT) is one of the most important virulence factors of *Pasteurella multocida* type D. *Pasteurella multocida* infection has caused enormous economic losses in the pig farming industry. Although it is well known that this bacterial infection causes progressive atrophic rhinitis, its effects on other organ tissues in pigs are unclear. In this study, PMT was expressed and purified, and the cytotoxic effects of PMT on four types of swine cells, LLC-PK1, PAM, IPEC, and ST, were investigated. LLC-PK1 exhibited the highest sensitivity to the cytotoxic effects of PMT. Our studies revealed that a PMT concentration of 0.1 μg/kg can lead to weight loss, whereas a PMT concentration of 0.5 μg/kg can lead to death in mice. PMT causes damage to the intestines, kidneys, lungs, livers, and spleens of mice. Furthermore, PMT caused acute death in pigs at treatment concentrations greater than 5 μg/kg; at PMT concentration of 2.5 μg/kg, weight loss occurred until death. PMT mainly caused damage to the hearts, lungs, livers, spleens and kidneys of pigs. The organ coefficient showed that damage to the heart and kidneys was the most severe and caused the renal pelvis and renal pyramid to dissolve and become cavitated. Pathology revealed hemorrhage in the lungs, liver, and spleen, and the kidneys were swollen and vacuolated, which was consistent with the damaged target organs in the mice. In conclusion, these findings demonstrate that PMT is extremely toxic *in vitro* and *in vivo*, causing damage to various organs of the body, especially the kidneys and lungs. This study provides a theoretical basis for the in-depth exploration of the cytotoxic effects of PMT on target organs.

## 1 Introduction

The small, nonmotile, facultatively anaerobic, gram-negative coccobacillus *Pasteurella multocida* (*P. multocida*) belongs to the *Pasteurellaceae* family (Wilkie et al., 2012; Wilson and Ho, 2013). *P. multocida* is an important zoonotic agent that causes a wide spectrum of diseases in many species of domestic and wild animals and even humans, including hemorrhagic septicemia (HS) in ungulates, fowl cholera (FC) in avian species, atrophic rhinitis (AR) in pigs, pneumonia in sheep and pigs, and snuffles in rabbits (Moustafa et al., 2015; Mbuthia et al., 2008; Lariviere et al., 1992; García-Alvarez et al., 2017; Digiacomo et al., 1991). Occasional zoonotic infections in humans have been reported (Fátima et al., 2018; Donnio et al., 1991). *P. multocida* strains are categorized into five serotypes according to their capsular antigens: A, B, D, E, and F (Carter, 1955). *P. multocida* toxin (PMT) is expressed by Type D strains and some Type A strains of *Pasteurella* and is responsible for porcine AR (Amigot et al., 1998; Rutter, 1983). PMT mainly affects the turbinate bones and lungs of pigs, causing a series of secondary infections that result in serious losses to the global pig industry (Truswell et al., 2023; Peng et al., 2019).

PMT, a potent mitogenic protein (Lax and Grigoriadis, 2001), can induce cell proliferation by activating the PI3K-AKT signaling pathway (Preu et al., 2010), G proteins Gq/11 and G12/13, and the Gi/o receptor pathway (Orth et al., 2013; Seo et al., 2000; Baldwin et al., 2003). For example, PMT can stimulate the differentiation of osteoclasts (Ebner et al., 2019; Sushmita et al., 2017) and Swiss 3 T3 cell proliferation (Oubrahim et al., 2012), activate ERK 1/2 and Akt phosphorylation in MLO-Y4 cells (Hannah et al., 2018) and antiapoptotic pathways in HEK293 cells (Preu et al., 2010) and increase the activity of NG108-15 neuronal cells (Surguy et al., 2014). Previous studies have reported that PMT mainly induces cell proliferation, causing a more significant toxic effect in bone cells (Kubatzky, 2022). PMT induces differentiation via Gαq/11-dependent activation of osteoclastogenesis (Hannah et al., 2018; Strack et al., 2014). In contrast, PMT inhibits the differentiation of osteoblasts through the activation of Gαq/11 and transactivation of MAPK signaling cascades (Siegert et al., 2013). Studies utilizing the PMT-DTa fusion protein-induced cell death model to investigate LRP1 as a potential PMT receptor (Schoellkopf et al., 2022) and employing immunoprecipitation to explore the potential receptors sphingomyelin (SM) and phosphatidylcholine (PC) (Brothers et al., 2011) have yielded incomplete results. This inconsistency is primarily attributed to the involvement of PMT in receptor binding, which involves both the N-terminus and C-terminus of PMT (Clemons et al., 2018; Bergmann et al., 2013; Kitadokoro et al., 2007). Moreover, several reports have described the effects of PMT on cell death. Previous studies have used guinea pigs as a model to evaluate these effects (Pennings and Storm, 1984). In addition, our previous research revealed that PMT induces PK15 cell death by regulating the *CXCL8* gene (Yuan et al., 2024). These studies indicate that the function of PMT is complex, and further exploration is warranted, especially for PMT-induced porcine cytotoxicity.

A previous study evaluated the systemic effects of *P. multocida* (Wilson and Ho, 2012). Immunohistochemical analysis of 30 cases of porcine dermatitis and nephropathy syndrome in pigs following brief clinical illness revealed *P. multocida*-specific staining in 26 cases. This staining was predominantly observed in the renal tubular epithelial cells of the proximal convoluted tubules, as well as in the glomeruli, renal vasculitis lesions, and the cytoplasm of interstitial mononuclear cells (Thomson et al., 2001). PMT can infect nasal turbinates directly when the toxin-producing strain is found in the nasal cavity of an animal. The toxins produced by *P. multocida* may also affect anatomical sites such as tonsils, where bacteria can colonize and subsequently spread throughout the body (Hamilton et al., 1998). In addition, several studies have shown that PMT causes myocardial fibrosis in mice (Weise et al., 2015) and weight loss and liver necrosis in rats (Cheville and Rimler, 1989). Previous studies have reported that PMT administered via intraperitoneal injection has a diverse range of tissue-and cell-specific *in vivo* effects in mice, including G-alpha protein modification, stimulation of proliferation markers, and expression of active β-catenin (Banu et al., 2020). More recently, PMT-induced pneumonia in mice has been reported (Xiao et al., 2023). Therefore, we investigated the systematic correlation between PMT-induced cytotoxicity *in vitro* and targeted organ damage in mice and pigs *in vivo*. In this study, as previously described (Yuan et al., 2024), PMT was initially expressed and subsequently assessed for its cytotoxic effects on various pig cell lines, including LLC-PK1, IPEC, ST, and PAM. Our findings indicate a significant cytotoxic impact of PMT on pig cells. Furthermore, the toxicological effects of PMT on mice were investigated, revealing a reduction in mouse weight, mortality and extensive organ damage at higher concentrations of PMT. This study subsequently assessed the toxic effects of PMT on pigs, revealing a correlation between increasing PMT concentrations and increasing toxicity levels, ultimately leading to significant organ damage. In particular, both *in vitro* and *in vivo* experiments revealed that the greatest toxic effects of PMT occur in the kidneys, causing severe renal vacuolization, kidney enlargement, and hydronephrosis. Histopathological analysis of renal tissues revealed increased congestion in the renal interstitium, multiple renal tubular dilatations, and hydropic degeneration in renal tubular epithelial cells, among other pathological changes. This comprehensive examination of both the *in vivo* and *in vitro* toxic effects of PMT lays the groundwork for further investigations into the pathogenic mechanisms of these toxic effects and has importance in the prevention and treatment of AR in pigs.

## 2 Materials and methods

### 2.1 Expression of the PMT recombinant protein

A recombinant His-tagged PMT or inactive PMT mutant (∆PMT) containing a C-terminal cysteine-to-serine alteration (C1165S) derived from *P. multocida* strain HN06 was expressed in *E. coli* (BL21 strain) by cloning with a pColdI vector and purified by Ni2+ affinity chromatography with Profinity™ IMAC Ni-Charged Resin (Bio-Rad, United States). The concentration of PMT was quantified by a BCA protein assay kit (Biomed, China). The purified PMT protein was subsequently dialyzed in a dialysis bag and concentrated in a 50 kDa ultrafiltration tube (Millipore, United States). The detailed steps of the expression of PMT and ∆PMT were performed as we previously reported (Yuan et al., 2024).

### 2.2 Cell culture

LLCPK (BNCC359398), ST (BNCC338128), PAM (BNCC338298), and IPEC (BNCC338252) cells were all purchased from BNCC. LLC-PK1, IPEC, and ST cells were cultured in Dulbecco’s modified Eagle’s medium (DMEM; Gibco, United States) supplemented with 0.37% sodium bicarbonate, 100 U/mL penicillin–streptomycin (Solarbio, China), and 10% fetal bovine serum (FBS; PNA, Germany) in a humidified atmosphere of 95% air and 5% CO2. PAM cells were cultured in RPMI 1640 (Gibco 31800-022) medium supplemented with 10% FBS in the presence of penicillin–streptomycin.

### 2.3 Cell viability assay

LLC-PK1, IPEC, PAM, and ST cells exhibiting superior growth characteristics were identified and seeded into 96-well plates at a density of 5,000 cells/150 μL. Upon reaching 90% confluence, the medium was replaced with PBS, and PMT at concentrations of 1 ng/mL, 10 ng/mL, 100 ng/mL, and 1,000 ng/mL was added to the experimental group, while normal cells were maintained as a blank control. The cells were incubated in a constant-temperature incubator (with 5% CO2) at 37°C for 48 h in serum-free DMEM or RPMI 1640 medium. Fifteen microliters of CCK-8 reagent (Meilun, Dalian, China) was dispensed into each well, followed by a 1-h incubation period at 37°C. Absorbance readings were taken at a wavelength of 450 nm using a microplate reader (Thermo Scientific, Waltham, MA, United States). Cell viability was calculated as [(mean OD450 treatment-mean OD450 blank)/(mean OD450 control-mean OD450 blank)] × 100%.

### 2.4 Mouse models

All the animal studies were conducted according to protocols approved by the Sichuan Agricultural University Ethics Committee (Approval Nos. SYXK 2019-187 and 20210035). C57BL/6 mice were purchased from Dossy (Chengdu, China). The mice were housed in cages with continuous access to food and water, with each cage accommodating a maximum of five female mice under controlled temperature (24°C) and humidity (55 ± 10%) conditions, with a 12 h light/dark cycle (lights on at 7:00 am). To prevent potential degradation of PMT by gastric acid and enzymes, the intraperitoneal (i.p.) route of administration was chosen. Consequently, in the PMT challenge experiments, male mice aged eight to ten weeks were randomly assigned to one of the experimental group or the control group. PMT doses of 0.1 and 0.5 μg/kg (body weight) were diluted in PBS and administered to the mice via the i.p. route. Buffer (PBS, 0.1% BSA) and ∆PMT alone were used as controls. All the PMT-challenged or infected mice were monitored three times daily for signs of malaise or mortality for ten days post challenge, and their weights were measured daily. Necropsies were performed on the deceased mice, all remaining mice were euthanized after 10 days, and necropsies were subsequently conducted. Organs such as the heart, liver, spleen, lungs, kidneys, and intestines were harvested from the mice and preserved in 4% paraformaldehyde for future analysis.

### 2.5 Swine models

All the animal studies were conducted according to ethical regulations under protocols approved by Sichuan Agricultural University (Approval Nos. SYXK 2019-187 and 20210035). The piglets were obtained from Sichuan Agricultural University. Pigs were housed in cages with 24-h free access to food and water, with each cage holding a maximum of three male pigs at a constant temperature (30°C) under a 12 h light/dark cycle (lights on at 7:00 am). Following a one-week period of acclimatization to the environment, all the piglets were allocated into experimental groups or control groups through random assignment. The piglets were then administered varying concentrations of PMT (1.5 μg/kg, 2.5 μg/kg, 5 μg/kg, and 10 μg/kg) via intramuscular injection into the neck, with the control group receiving physiological saline. Signs of malaise or mortality were monitored and recorded daily, and regular weighing was conducted throughout the 14-day feeding period. All the piglets were euthanized and dissected to obtain select organs and tissues, including the heart, liver, spleen, lungs, and kidneys, for the weighing and photographic documentation to calculate the organ coefficient. Additionally, fresh organ tissues were selected and fixed with 4% paraformaldehyde for subsequent pathological sectioning and histological analysis by hematoxylin and eosin(H&E) staining.

### 2.6 Organ coefficient

Prior to dissection, the deceased piglets were weighed, and their weight were recorded. Various organs, including the heart, liver, spleen, lungs, and kidneys, were subsequently extracted from piglets. Each organ was cleaned with physiological saline, surface moisture was absorbed using standard paper towels, the samples were weighed, and the weight of each organ was recorded. Organ coefficients (e.g., heart coefficient, liver coefficient, kidney coefficient, spleen coefficient and lung coefficient) were calculated by dividing the net weight of each organ by the body weight of the respective piglet.

### 2.7 H&E staining

Freshly harvested mouse and pig tissues were fixed with 4% formalin in phosphate buffer. The tissues were rinsed with running water for 30 min, subjected to antigen retrieval, and subsequently placed in a pathological embedding plastic basket for dehydration via a series of ethanol solutions (75% ethanol for 6 h, 85% ethanol for 10 h, 95% ethanol for 4 h, anhydrous ethanol for 12 h, and anhydrous ethanol for 2 h). The samples were then subjected to transparency treatment (xylene I for 20 min, xylene II for 15 min) and immersion in wax for 3 h, and the tissue block was finally embedded in paraffin. The tissue was subsequently sectioned into 5 μm thin slices using a slicer (RM2235, Leica, Germany), flattened in warm water, transferred onto a glass slide, and baked at 60°C for 2 h. Following dewaxing with xylene, the tissue slices underwent washing with running water for 20 min, staining with hematoxylin (Thermo Scientific, Waltham, MA, United States) for 30 min, further washing for 20 min, differentiation with hydrochloric acid alcohol, staining with eosin (Thermo Scientific, Waltham, MA, United States) for 5 min, dehydration with gradient alcohol, transparency treatment with xylene, and sealing with resin adhesive. The tissue was subsequently examined under a microscope to observe pathological changes, and a microscopic imaging system (DM1000, Leica, Germany) was used to capture images and document normal tissue or areas displaying evident lesions.

### 2.8 Statistical analysis

Body weight data from the mice and pigs are presented as the means ± standard deviations (SDs). Analysis of variance (ANOVA) with *post hoc* tests was used to assess significant differences between two groups, and survival curves were compared using the log-rank Mantel–Cox test with GraphPad Prism 9 software (GraphPad Software, Inc., San Diego, CA, United States). Differences were considered statistically significant if the *p* value was <0.05.

## 3 Results

### 3.1 Cytotoxic effects of PMT on pig cells

**Figure 1:**
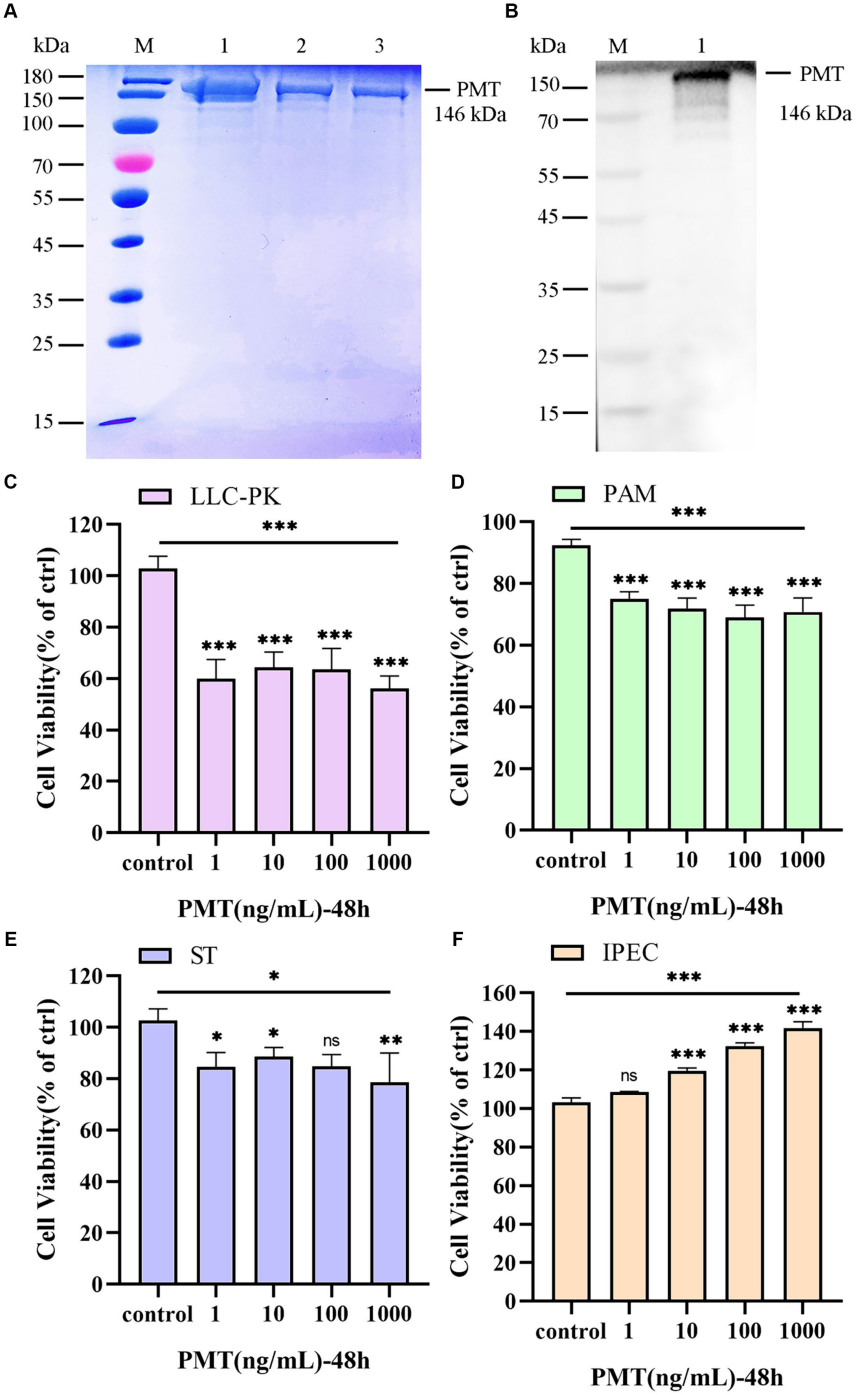
Expression of PMT and analysis of its *in vitro* cytotoxicity. **(A)** Expression of PMT. M, protein marker; lines 1–3, recombinant His-tagged PMT. **(B)** Western blotting verification of the PMT protein. M, protein marker; line 1, recombinant His-tagged PMT. **(C)** CCK-8 assay of the viability of LLC-PK1 cells after incubation with 1, 10, 100 or 1,000 ng/mL PMT for 48 h. **(D)** CCK-8 assay of the viability of PAM cells treated with different concentrations of PMT for 48 h. **(E)** CCK-8 assay of ST cells after incubation with different concentrations of PMT for 48 h. **(F)** CCK-8 assay of ST cells after incubation with different concentrations of PMT for 48 h. Data are presented as the means ± SDs *n* = 3. ns > 0.05, * *p* < 0.05, ** *p* < 0.01, *** *p* < 0.001.

We investigated the cytotoxicity of PMT in porcine cells *in vitro*. As we previously reported (Yuan et al., 2024), the expected molecular mass of the PMT protein was confirmed via SDS–PAGE (Figure 1A, lines 1–3). Consistent with the SDS–PAGE results, Western blotting analysis with monoclonal anti-His antibodies verified the expression of PMT (Figure 1B, line 1). Varying concentrations of PMT elicited differential cytotoxic responses across distinct cell types. Compared with PAM, ST and IPEC cells, LLC-PK1 cells became rounded and died in significant amounts after treatment with PMT at a concentration of 1 ng/mL (Supplementary Figure S1). LLC-PK1 cells exhibited significant decreases in viability following 48 h of exposure to a PMT concentration of 1 ng/mL, as determined by the CCK-8 assay (Figure 1C). However, further increases in the PMT concentration did not result in an augmented cytotoxic effect on LLC-PK1 cells (Figure 1C and Supplementary Figure S1). Similarly, various concentrations of PMT exerted a range of cytotoxic effects on PAM and ST cells after 48 h of exposure (Figures 1D,E). In contrast, PMT treatment had a dose-dependent effect on cell proliferation only in IPEC cells; as the concentration of PMT increased, the effects on cell proliferation became more pronounced (Figure 1F). The observed cytotoxicity results suggest that PMT expression results in substantial toxicity and robust activity *in vitro*. Consequently, the toxicity of PMT was investigated *in vivo*.

### 3.2 Clinical characteristics of PMT toxicity in mice

**Figure 2:**
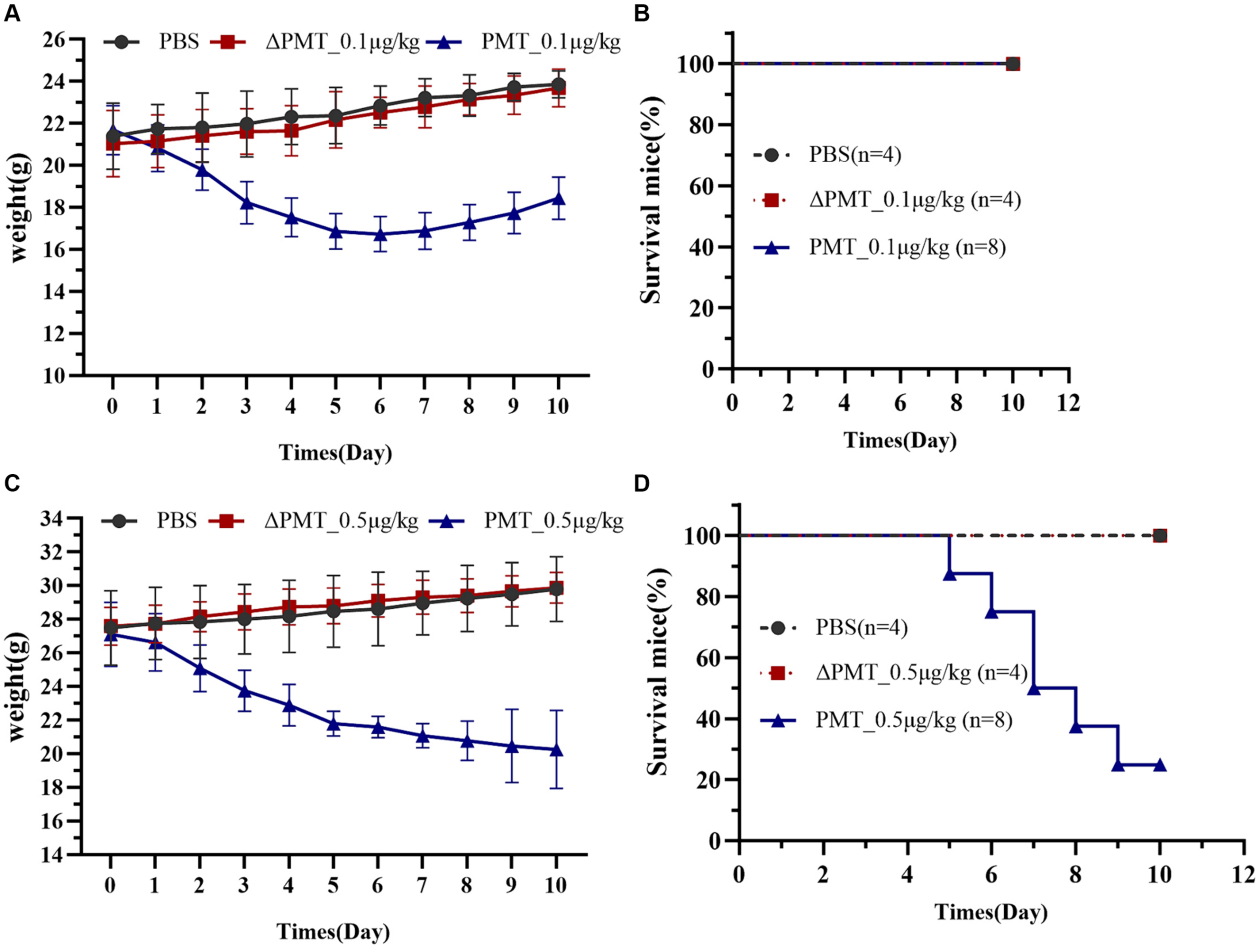
Effect of PMT treatment on weight gain in C57BL/6 mice. Effects of low-dose and high-dose PMT treatment in mice *in vivo*. The mice were treated with 0.1 μg/kg or 0.5 μg/kg (body weight) recombinant PMT, ∆PMT (∆PMT; 0.1 μg/kg or 0.5 μg/kg), or PBS for 10 days as indicated. Body weights were measured and are depicted as **(A)** actual weights from 0 to 10 days (A, PMT; 0.1 μg/kg) and **(B)** the percentage of living mice after 0 to 10 days of incubation with 0.1 μg/kg PMT. **(C)** Actual weights of the mice from 0 to 10 days after incubation with 0.5 μg/kg PMT. **(D)** The percentage of living mice from 0 to 10 days after incubation with 0.5 μg/kg PMT. The data are presented as the means ± SDs (*n* = 4–8 per group).

To evaluate the toxicological impact of PMT on various organs and tissues in mice, a low concentration of PMT (0.1 μg/kg) was administered via i.p. injection. The inactive mutant ΔPMT (containing a mutation of cysteine-to-serine at the 1165th amino acid position of PMT) and PBS were employed as negative controls. The mice were continuously monitored, and their body weights were recorded daily. Observations revealed a decrease in overall activity levels and a gradual reduction in body weight after PMT treatment. After the fifth day, the weights of the mice did not decrease and gradually increased over time (Figure 2A). Although no mortality was observed, the findings suggested that a dose of 0.1 μg/kg PMT had toxic effects on the mice (Figure 2B). We subsequently increased the dose of PMT. Injection of 0.5 μg/kg PMT into the abdominal cavity of mice resulted in a significant reduction in body weight (Figure 2C). Mortality was first observed by the fifth day, and a progressive decline in survival was noted over time (Figure 2D). In contrast, the mice in the control groups, which included the ΔPMT and PBS groups, exhibited no weight loss (Figure 2D). These findings indicate that PMT has a substantial toxic effect on mice.

### 3.3 Organ damage caused by PMT in mice

**Figure 3:**
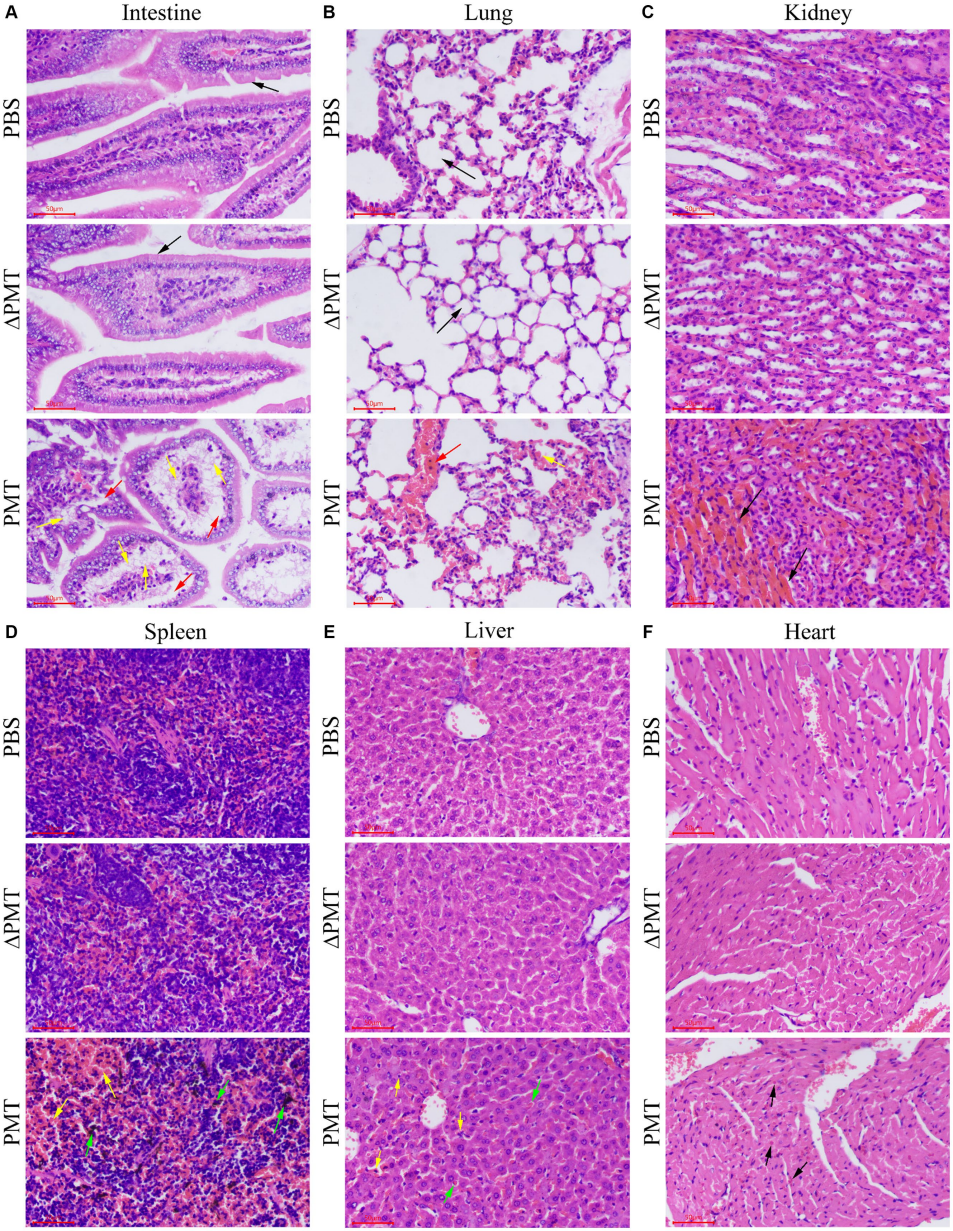
Pathological damage to mouse organs after treatment with PMT. Hematoxylin and eosin (H&E) was used to stain the intestines, lungs, kidneys, spleens, livers and hearts of the mice after i.p. injection with 0.5 μg/kg PMT (body weight), 0.5 μg/kg ∆PMT (body weight) or PBS. **(A)** The normal and damaged intestinal tissues are marked with different colored arrows. **(B)** The normal and damaged lung tissues are indicated with different colored arrows. **(C)** Damaged kidney tissue is marked with black arrows. **(D)** Damaged spleen tissue is marked with different colored arrows. **(E)** The damaged liver tissue was marked with black and green arrows. **(F)** Damaged heart tissue is denoted with black arrows. Scale bar, 50 μm.

To further investigate the impact of PMT on various organs in mice, we dissected and collected samples from mice that were administered 0.5 μg/kg PMT. Pathological staining of organ tissues was subsequently performed. In the control groups, the epithelial cells within the mucosal layer exhibit an intact architecture, with no signs of damage, and the villi were distinctly visible, orderly and devoid of any pathological alterations (black arrows). Compared with the PBS control group, the PMT-exposed group showed significant intestinal villus shedding, severe cellular steatosis (yellow arrows), and extensive infiltration of adipocytes into the mucosal lamina propria of the small intestine, which resulted in the disappearance and partial detachment of the epithelium. The mucosal layer of the intestine exhibited looseness and swelling (red arrows) (Figure 3A and Supplementary Figure S2A). In the PBS control group, no red blood cells were found (black arrows) within the alveolar spaces, and the lung tissue appeared normal. Conversely, the mice in the treatment group exhibited mild pulmonary hemorrhage, characterized by the presence of a small quantity of erythrocytes (yellow arrows) and blood vessel congestion (red arrows) within the alveolar spaces (Figure 3B and Supplementary Figure S2B). Additionally, renal histopathology revealed increased congestion in the renal interstitium (black arrows) in the PMT treatment group (Figure 3C and Supplementary Figure S2C). The demarcation between the red and white pulp of the spleen was distinct, with the splenic sinuses in the red pulp region appearing enlarged and congested (yellow arrows). Localized hemorrhaging was evident, accompanied by substantial deposition of brown pigment (green arrows). Numerous nuclear fragments and dissolution were present within the lymphoid follicles in the spleen (yellow arrows) (Figure 3D and Supplementary Figure S2D). The liver showed mild hemorrhaging (black arrows) (Supplementary Figure S2E), and a minor accumulation of red blood cells was observed within the hepatic sinusoids (green arrows). Additionally, there was occasional karyopyknosis and deep staining of hepatocyte nuclei, and increased eosinophilia, accompanied by a modest infiltration of neutrophils (yellow arrows) (Figure 3E and Supplementary Figure S2E). No significant pathological alterations were observed in the cardiac tissue in the PBS control group, and a minor accumulation of red blood cells was detected in the heart after with PMT treatment (black arrows) (Figure 3F and Supplementary Figure S2F). As a negative control group, ΔPMT-exposed mice presented no significant pathological alterations in their tissues (Figure 3 and Supplementary Figure S2). These findings suggested that PMT induced varying degrees of damage across multiple organs in mice, with the small intestine, which was directly exposed to PMT, showing the most pronounced damage. Additionally, organs such as the lungs, kidneys, liver, and spleen were affected to different extents.

### 3.4 *In vivo* effects of PMT toxicity on pigs

**Figure 4:**
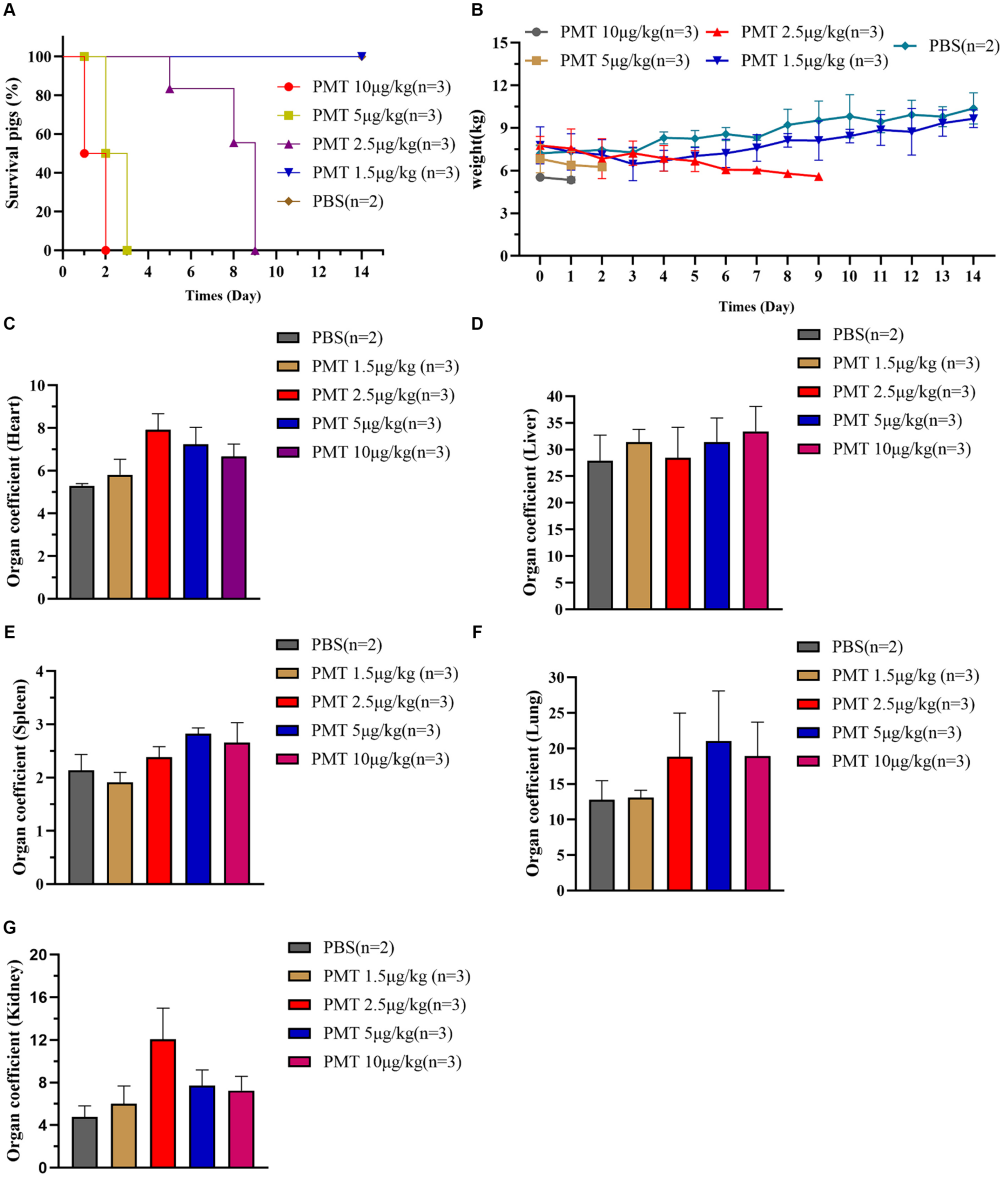
Lethal toxicity of PMT in piglets. **(A)** Weaned WT piglets were treated with different concentrations of PMT via intramuscular injection, and PBS-treated piglets served as the control. The piglets were continuously observed for 14 days, piglet mortality was recorded, and the survival rate was calculated. **(B)** After different doses of PMT were administered to piglets, the weights of the piglets were recorded and analyzed within 14 days. **(C–G)** After 14 days of continued feeding, all piglets were subsequently euthanized, piglet organ tissues were removed and weighed, and their organ coefficients were calculated and analyzed. **(C)** Heart coefficient, **(D)** liver coefficient, **(E)** spleen coefficient, **(F)** lung coefficient, and **(G)** kidney coefficient. The data are presented as the means ± SDs (*n* = 2–3).

According to the above findings, PMT had notable cytotoxic effects on various cell types, including pig kidney cells, lung cells, intestinal epithelial cells, and testicular cells. Furthermore, PMT resulted in substantial organ damage in mice, including damage to the kidneys, lungs, spleen and intestines. To expand upon those results, we sought to investigate the toxic effects of PMT on pigs. Varying concentrations of PMT were administered randomly into the neck muscles of weaned piglets. At concentration of 5 μg/kg and 10 μg/kg, all piglets died within 3 days. Compared with the PBS control group, acute toxicity was observed in piglets exposed to PMT at a concentration of 2.5 μg/kg, resulting in mortality, whereas no deaths occurred at a concentration of 1.5 μg/kg (Figure 4A). The piglets treated with 2.5 μg/kg PMT exhibited progressive weight less over time, and the piglets treated with 1.5 μg/kg PMT had lower weights than the control piglets did. Over time, the weights of the piglets gradually normalized (Figure 4B). Following euthanasia, the piglets were dissected, and the weights of their hearts, livers, spleens, lungs, and kidneys were recorded. Owing to the limited sample size of the piglets, differential analysis could not be performed, but the data revealed an obvious increase in the heart coefficient with increasing PMT dose (Figure 4C). As the dose of PMT increased, the liver coefficient increased correspondingly (Figure 4D). The spleen coefficient slightly increased (Figure 4E), and the lung coefficient also demonstrated an upward trend (Figure 4F). In contrast, the kidney coefficient initially increased but subsequently decreased with increasing PMT challenge dose, reaching its peak value at a dose of 2.5 μg/kg (Figure 4G). These findings indicate that piglets are highly likely to die from PMT-induced damage to various tissues and organs.

### 3.5 Organ damage caused by PMT in pigs

**Figure 5:**
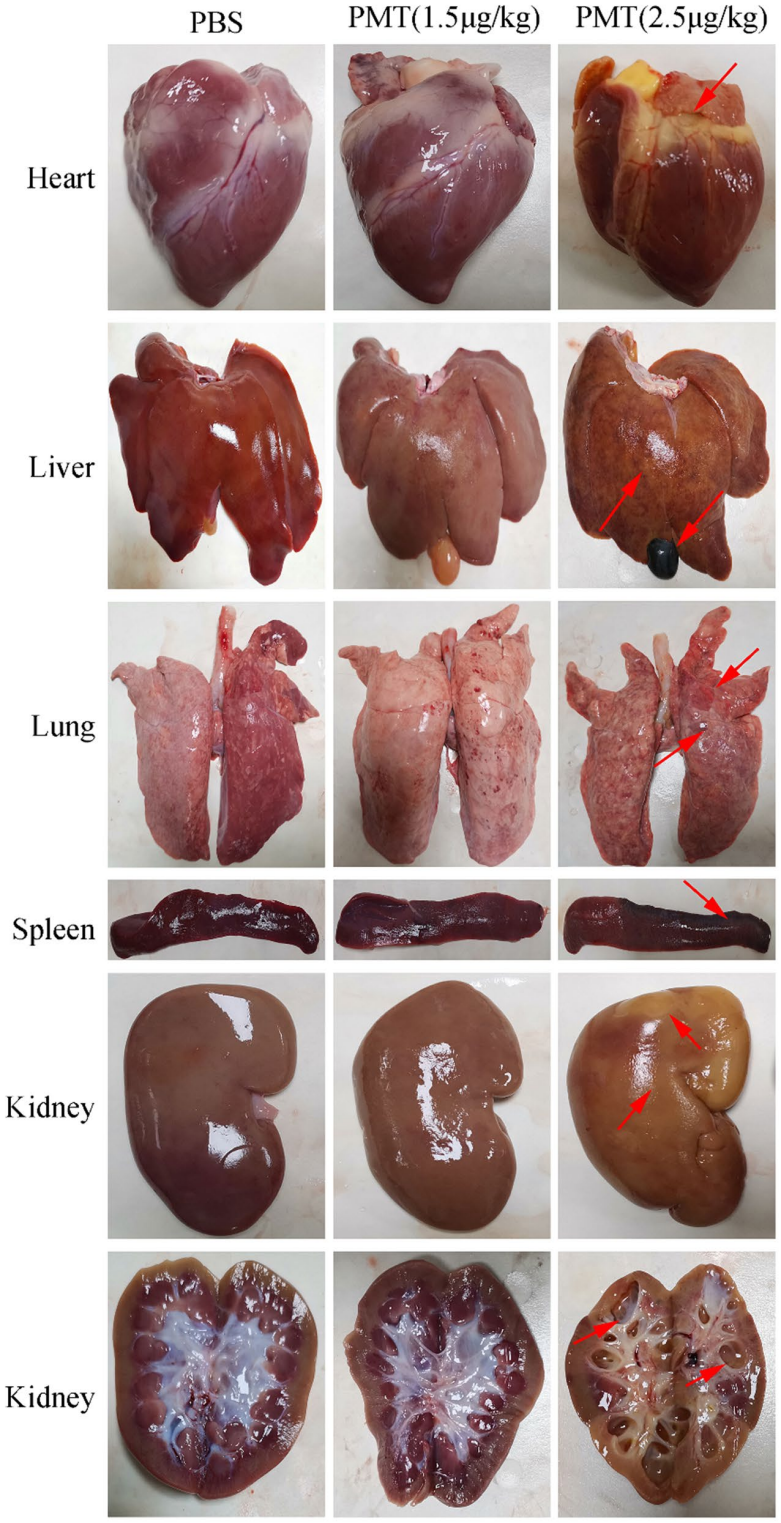
Toxic effects of PMT on piglet organs. The piglets were administered different concentrations of PMT. The piglets exposed to 2.5 μg/kg (body weight) PMT were promptly dissected after death, whereas those exposed to 1.5 μg/kg (body weight) PMT and the PBS control treatment were dissected after a 14-day period. Organs, including the heart, liver, spleen, lung, and kidney, were extracted for photographic documentation, and organ damage is indicated with red arrows. Transverse images of the renal tissue were captured to assess the extent of damage to the renal pelvis, renal cortex, and renal medulla (red arrows).

Photographs were taken of the organs of the dissected piglets, and the PMT-treated pigs were compared with those of the PBS control piglets. Following the administration of PMT to piglets at dosages of 5 μg/kg and 10 μg/kg, post-mortem examinations revealed varying degrees of organ damage across all the samples. The skin of the infected piglets clearly exhibited jaundice, and post-mortem examinations revealed pronounced yellow discoloration in the heart and liver of piglets exposed to 5 μg/kg PMT (Supplementary Figure S3). These findings indicated that the piglets succumbed to multiple organ failure induced by PMT. We subsequently analyzed organ damage in piglets treated with 2.5 μg/kg and 1.5 μg/kg PMT. Following the administration of 2.5 μg/kg PMT, the piglets displayed symptoms of cardiac bleeding and jaundice, along with liver bleeding and lung damage, as indicated by the red arrows. The spleen exhibited brown deposits, with the most notable findings being kidney enlargement, severe vacuolization, and hydronephrosis. Anatomical examination revealed significant damage to the renal pelvis, as indicated by the red arrows (Figure 5). When piglets were administered PMT at dosages of 5 μg/kg and 10 μg/kg, acute mortality was observed. The clinical manifestations of poisoning and organ damage were analogous to those observed in piglets administered 2.5 μg/kg PMT, except that the onset of symptoms was delayed in piglets treated with the lower dose. Conversely, when PMT was administered at a dose of 1.5 μg/kg, the piglets exhibited slight liver swelling and brown pigment deposition in the spleen, whereas no significant changes in other organs were observed (Figure 5).

**Figure 6:**
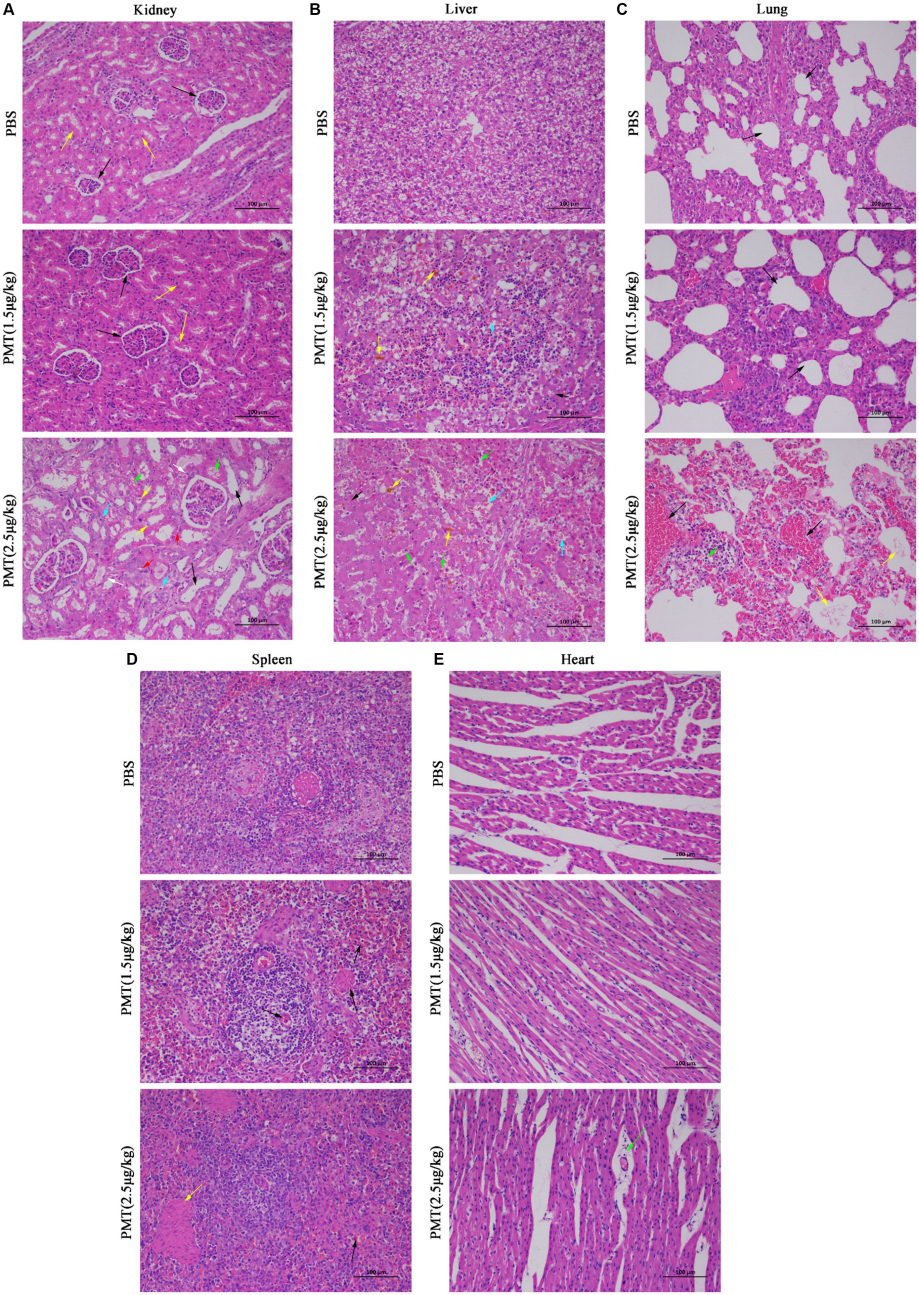
H&E pathological staining of organs and tissues in pigs. Hematoxylin and eosin (H&E) was used to stain the lungs, kidneys, spleens, livers and hearts of piglets after injection with PMT at 1.5 μg/kg or 2.5 μg/kg (body weight) or PBS after 14 days. **(A)** The normal and damaged kidney tissues are marked with distinct colored arrows. **(B)** Damaged liver tissue is identified with various colored arrows. **(C)** The normal and damaged lung tissues are indicated with different colored arrows. **(D)** Damaged spleen tissue is denoted with black and yellow arrows. **(E)** Damaged heart tissue is marked specifically with green arrows. Scale bar, 100 μm.

Following the administration of 2.5 μg/kg and 1.5 μg/kg PMT to the piglets, H&E staining was conducted on their organs and tissues. Piglets exposed to 2.5 μg/kg PMT exhibited significant organ damage. Specifically, compared with those in the control group, the renal tissue of PMT-treated piglets exhibited several pathological alterations: renal tubular dilation (black arrows), widespread renal tubular atrophy (green arrows), the lumen of the renal tubule showed narrowing or even complete obliteration (red arrows); numerous of hydropic degeneration of renal tubular epithelial cells, characterized by loosely and pale stained cytoplasm (yellow arrows); and increased connective tissue with small amount of lymphocytic infiltration (white arrows). Minor renal tubular epithelial cell necrosis with karyopyknosis (blue arrows) occurred (Figure 6A and Supplementary Figure S4A). However, compared with those in the PBS control group, in the piglets exposed to 1.5 μg/kg PMT, in the renal cortex, the glomeruli were evenly distributed, indicating a consistent and uniform the cellular and matrix components of the glomeruli (black arrows), the epithelial cells of the renal tubules were characterized by their rounded and full morphology, with brush borders that are orderly and regularly arranged (yellow arrows); the renal medulla demonstrates no observable abnormalities, and there was no significant hyperplasia in the interstitium. Additionally, no significant inflammatory changes were detected (Figure 6A and Supplementary Figure S4A). After PMT treatment, liver cells exhibited significant damage characterized by steatosis in hepatic cells (black arrows), hepatocellular necrosis, karyopyknosis and fragmentation (green arrows), increased eosinophilia, congestion and dilation of hepatic sinusoids (blue arrows), and minor deposition of lipofuscin (yellow arrows) (Figure 6B and Supplementary Figure S4B). The lungs exposed to 2.5 μg/kg PMT displayed pronounced hemorrhage, edema (black arrows), widened alveolar septa, a few fibrillar protein deposition in the alveolar spaces (yellow arrows). Notably, a substantial presence of red blood cells, neutrophils (green arrows), and alveolar macrophages was observed, and piglets exposed to 1.5 μg/kg PMT exhibited mild pulmonary congestion and a minimal presence of erythrocytes (Figure 6C and Supplementary Figure S4C). With PMT treatment, an abundance of lymphocytes was observed in the white pulp of spleen (black arrows), along with a substantial presence of spleen trabecula in the red pulp (yellow arrows), with no significant abnormalities detected overall (Figure 6D and Supplementary Figure S4D). No significant alterations were noted in the heart relative to the animals in the control group, and the myocardial fibers exhibited a slightly loose arrangement, accompanied by punctate lymphocyte infiltration (green arrows) (Figure 6E and Supplementary Figure S4E). These findings unequivocally illustrate the pronounced toxic impact of PMT on porcine physiology, resulting in diverse types of organs and tissue damage. This study provides initial documentation of PMT-induced toxicity in pigs, highlighting its deleterious effects on multiple organs that ultimately lead to fatal outcomes.

## 4 Discussion

An important toxin produced by *P. multocida*, PMT primarily induces nasal turbinate atrophy (To et al., 2005). Most previous studies have concentrated on the transport of the PMT protein and its role in inducing cell proliferation (Repella et al., 2011; Hildebrand et al., 2015). However, there are also reports that PMT promoted cell death in PK15 pig kidney cells (Yuan et al., 2024), suggesting that PMT exhibits varying forms of toxicity across different cell types. Our findings demonstrate that PMT is highly toxic to porcine cells, specifically LLC-PK1 cells, which are renal proximal tubular epithelial cells. These cells exhibited significant cell death upon PMT stimulation, underscoring the strong cytotoxic effects of PMT. Additionally, the PAM and ST cells also exhibited a degree of cell death when treated with PMT. However, only IPEC cells exhibited significant cell proliferation under PMT stimulation. These results underscore the distinct cell-specific toxicity of PMT, highlighting its potential for targeted porcine renal cell death. Although we did not further investigate the mechanism underlying the death of LLC-PK1 cells, screening for the PMT receptor on basis of its toxic effects on porcine renal cells may yield novel insights. The lack of emphasis on non-bone cell targets and the absence of a reliable model for PMT-induced cell lethality has hindered the identification of key PMT receptors in host cells, underscoring the importance of further research in this area.

Turbinate atrophy, the primary manifestation of PMT toxicity, is a common research focus (Williams et al., 1990; Hsuan et al., 2009), and the nasal bone is a well-established target organ of PMT *in vivo* because of its causal relationship with AR. Other studies have demonstrated that PMT also mediates proliferation in pig bladder epithelium (Hoskins et al., 1997), induces liver and kidney damage in pigs and rats (Rutter and Mackenzie, 1984; Cheville and Rimler, 1989), and causes hypertrophy of mouse heart tissue (Weise et al., 2015). These findings suggest that, in addition to bone tissue, PMT may have additional *in vivo* targets. However, the cytotoxic effects of PMT on multiple organs have been overlooked. Comprehensive investigations into the *in vitro* cytotoxicity and *in vivo* target organ toxicity of PMT are lacking. Although mice are frequently employed as animal models for pathogen research, discrepancies may still exist between studies conducted on mice and those conducted on host pigs. Notably, pigs are highly susceptible to *P. multocida*, with PMT predominantly targeting the nasal bones of pigs. A small number of studies have reported that PMT may induce nasal turbinate atrophy in piglets (Dominick and Rimler, 1986; Horiguchi, 2012); however, the effects on their internal organs remain poorly understood. To address this, we exposed both mice and piglets to varying concentrations of PMT to comprehensively assess its damaging effects on target organs in different organisms. Given that PMT is a protein toxin, administration can only be achieved via injection. Intraperitoneal injection of 0.1 μg/kg PMT in mice did not result in mortality but did impact growth and weight, with weight gradually increasing as the mice developed tolerance. This finding is consistent with the results of PMT administration via intramuscular injection at a dosage of 1.5 μg/kg in piglets, wherein an initial decline in body weight was noted, followed by a gradual recovery over time. These findings demonstrate that low-dose PMT can cause weight loss (Banu et al., 2020). Several studies have shown that the subcutaneous injection of PMT in rats results in significant hepatic injury and growth inhibition (Cheville and Rimler, 1989). These findings are consistent with observations of PMT toxicity in mice and piglets. However, in addition to hepatic damage, we also observed concurrent injury to the lungs and kidneys. It was speculated that since the liver is the primary metabolic organ, it is susceptible to varying degrees of damage from most toxins (Zhu et al., 2021; Kessoku et al., 2021; Cai et al., 2024). Additionally, the lungs, the primary target organ of *P. multocida* (Peng et al., 2017), exhibit a certain degree of pathological damage.

The administered dose of PMT was subsequently increased. Mice that were challenged intraperitoneally at a dose of 0.5 μg/kg exhibited both weight loss and mortality. Similarly, piglets subjected to an increased dosage of 2.5 μg/kg not only experienced weight loss but also demonstrated significant mortality. Pathological examination of the anatomical tissues from deceased mice and piglets revealed varying degrees of organ damage, with the most severe damage observed in the intestines of mice. This pronounced intestinal damage is likely attributable to the intraperitoneal injection of PMT. Examination of the organs of the piglets, showed that all organs exhibited varying degrees of damage. Pathological staining confirmed that multiple organ tissues were affected to different extents, with the liver and kidneys being the most severely compromised. This phenomenon could be because PMT, as an exogenous protein, undergoes metabolism and degradation in the body, resulting in heightened damage to metabolic organs (Xiao et al., 2023; Meng et al., 2021; Guo et al., 2024). These observations corroborate previous research indicating that PMT can induce organ damage in animals (Hennig et al., 2008; Chrisp and Foged, 1991; Rajeev et al., 2003). Furthermore, immunohistochemical analysis of pigs afflicted with kidney disease demonstrated that *P. multocida* was predominantly localized within the renal tubular epithelial cells adjacent to the tubules. Additionally, the bacterium was detected in the cytoplasm of glomeruli, within renal vasculitis lesions, and among interstitial monocytes (Thomson et al., 2001). Critical observation via pathological analysis of damaged organ tissues from mice and pigs revealed that the kidneys and lungs were the most severely affected organs. Notably, these organs exhibited pronounced renal damage and pulmonary hemorrhage compared with other tissues. This finding is consistent with the substantial cell death in LLC-PK1 and PAM cells induced by PMT *in vitro*. Therefore, the kidney is a potential target organ for PMT. Furthermore, the administration of higher doses of 5 μg/kg and 10 μg/kg PMT to piglets resulted in acute poisoning, leading to mortality. Upon dissection, notable yellow discoloration was observed in the hearts and livers of piglets exposed to 2.5 μg/kg and 5 μg/kg PMT. The literature indicates that poisoning in piglets can result in hepatic metabolic damage, subsequently leading to jaundice (Wang et al., 2023; Osuna et al., 1982).

This study is subject to certain limitations, primarily due to the constraints associated with large-scale animal experiments and the challenges in precisely quantifying the levels of PMT toxins secreted by *P. multocida* in pigs. Our study included only 2–3 piglets per group, which is insufficient for conducting a robust difference analysis; however, as large experimental animals, pigs can yield valuable insights even with a limited sample size. Referencing similar studies with small sample sizes (Wang et al., 2021; Chen et al., 2023) and pertinent research findings showed that the intramuscular administration of 4 μg/kg PMT expressed *in vitro* replicated the symptoms of nasal nail bone atrophy in piglets infected with *P. multocida* (To et al., 2005). For a more comprehensive analysis of the piglet challenge experiments, we selected PMT dosages ranging from 10 μg/kg to 1.5 μg/kg. Notably, the piglets receiving a 2.5 μg/kg dose or greater died, whereas those receiving the 1.5 μg/kg dose survived; however, those piglets presented significant clinical symptoms. Although the administered PMT dosage was lower than that reported in the literature, it nonetheless resulted in mortality and exacerbated tissue damage. This phenomenon is likely attributable to advancements in protein expression and purification technologies, which have significantly improved the purity of the expressed PMT protein (Xiao et al., 2023; Yuan et al., 2024). Given the inherent challenges in precisely quantifying the concentration of PMT secreted by a body infected with *P. multocida*, on the basis of relevant research findings that our results were solely confined to the mortality and organ damage in piglets induced by PMT, with no manifestation of symptoms analogous to those engendered by *P. multocida* infection, particularly the characteristic nasal nail bone atrophy and alterations of facial morphology in pigs (Sakano et al., 1992; Chung et al., 1990; Ackermann et al., 1991). Therefore, we can only infer, the concentration of PMT secreted *in vivo* may be approximately 1.5 μg/kg or even less. Low concentrations of PMT were found to induce weight loss and hinder growth in mice and piglets, whereas high concentrations resulted in mortality.

In conclusion, the importance of this research lies in the systematic demonstration of the ability of PMT to induce specific toxic effects on renal cells and tissues, thereby establishing a model for PMT-induced renal cell death toxicity. This model can facilitate the identification of key PMT receptors and the analysis of the interaction between PMT and host receptors, which will be the focus of our future investigations. Our study systematically examined the distinct toxic effects of PMT on various cell types and organ tissues using both cellular and animal models. Our findings elucidated the deleterious impact of PMT *in vitro*, which are consistent with the organ damage observed *in vivo* for the first time. Acute toxicity of PMT most significantly harms to the kidneys and lungs, leading us to hypothesize that the kidney and lungs may serve as the principal target organs of PMT. These results establish a framework for future investigations into the toxicity mechanisms of PMT and offer a theoretical basis for the development of targeted therapies for PMT-related drugs. Additionally, our study provides a reference point for further research on the *in vivo* toxicity of different exogenous proteins.

## Data Availability

The original contributions presented in the study are included in the article/Supplementary material, further inquiries can be directed to the corresponding authors.

## References

[ref1] Ackermann M. R. Rimler R. B. Thurston J. R. (1991). Experimental model of atrophic rhinitis in gnotobiotic pigs. Infect. Immun. 59, 3626–3629. doi: 10.1128/iai.59.10.3626-3629.1991 1894365 PMC258930

[ref2] Amigot J. A. Torremorell M. Pijoan C. J. (1998). Evaluation of techniques for the detection of toxigenic *Pasteurella Multocida* strains from pigs. J. Vet. Diagn. Invest. 10, 169–173. doi: 10.1177/104063879801000209 9576345

[ref3] Baldwin M. R. Pullinger G. D. Lax A. J. (2003). *Pasteurella multocida* toxin facilitates inositol phosphate formation by bombesin through tyrosine phosphorylation of G alpha q. J. Biol. Chem. 278, 32719–32725. doi: 10.1074/jbc.M303524200 12799383

[ref4] Banu A. Lax A. J. Grigoriadis A. E. (2020). In vivo targets of *Pasteurella Multocida* toxin. Int. J. Mol. Sci. 21:2739. doi: 10.3390/ijms21082739, PMID: 32326543 PMC7215291

[ref5] Bergmann S. Jehle D. Schwan C. Orth J. H. Aktories K. (2013). *Pasteurella multocida* toxin as a transporter of non-cell-permeating proteins. Infect. Immun. 81, 2459–2467. doi: 10.1128/IAI.00429-13, PMID: 23630953 PMC3697606

[ref6] Brothers M. C. Ho M. Maharjan R. Clemons N. C. Bannai Y. Waites M. A.. (2011). Membrane interaction of *Pasteurella multocida* toxin involves sphingomyelin. FEBS J. 278, 4633–4648. doi: 10.1111/j.1742-4658.2011.08365.x, PMID: 21951695 PMC3220749

[ref7] Cai P. Liu S. Tu Y. Shan T. (2024). Toxicity, biodegradation, and nutritional intervention mechanism of zearalenone. Sci. Total Environ. 911:168648. doi: 10.1016/j.scitotenv.2023.168648, PMID: 37992844

[ref8] Carter G. R. (1955). Studies on *Pasteurella multocida*. I. A hemagglutination test for the identification of serological types. Am. J. Vet. Res. 16, 481–484. 13238757

[ref9] Chen R. Wen Y. Yu E. Yang J. Liang Y. Song D.. (2023). Identification of an immunodominant neutralizing epitope of porcine Deltacoronavirus spike protein. Int. J. Biol. Macromol. 242:125190. doi: 10.1016/j.ijbiomac.2023.125190, PMID: 37276902

[ref10] Cheville N. F. Rimler R. B. (1989). A protein toxin from *Pasteurella multocida* type D causes acute and chronic hepatic toxicity in rats. Vet. Pathol. 26, 148–157. doi: 10.1177/030098588902600208 2711572

[ref11] Chrisp C. E. Foged N. T. (1991). Induction of pneumonia in rabbits by use of a purified protein toxin from *Pasteurella multocida*. Am. J. Vet. Res. 52, 56–61. doi: 10.2460/ajvr.1991.52.01.56, PMID: 1826990

[ref12] Chung W. B. Bäckström L. R. Conrad T. Collins M. T. (1990). A comparison of different challenge methods for induction of atrophic rhinitis in pigs. APMIS 98, 442–452. doi: 10.1111/j.1699-0463.1990.tb01056.x, PMID: 2357344

[ref13] Clemons N. C. Bannai Y. Haywood E. E. Xu Y. Buschbach J. D. Ho M.. (2018). Cytosolic delivery of multidomain cargos by the N terminus of *Pasteurella multocida* toxin. Infect. Immun. 86:e00248-18. doi: 10.1128/IAI.00248-18 29784857 PMC6056849

[ref14] Digiacomo R. F. Allen V. Hinton M. H. (1991). Naturally acquired *Pasteurella multocida* subsp. multocida infection in a closed colony of rabbits: characteristics of isolates. Lab. Anim. 25, 236–241. doi: 10.1258/002367791780808365 1921322

[ref15] Dominick M. A. Rimler R. B. (1986). Turbinate atrophy in gnotobiotic pigs intranasally inoculated with protein toxin isolated from type D *Pasteurella multocida*. Am. J. Vet. Res. 47, 1532–1536, PMID: 3740622

[ref16] Donnio P. Y. Avril J. L. Andre P. M. Vaucel J. (1991). Dermonecrotic toxin production by strains of *Pasteurella multocida* isolated from man. J. Med. Microbiol. 34, 333–337. doi: 10.1099/00222615-34-6-333 2056517

[ref17] Ebner J. K. K. Kostenis G. M. Evisiegert P. Klausorth J. H. C. (2019). Activation of G(q) signaling by *Pasteurella multocida* toxin inhibits the osteoblastogenic-like actions of Activin A in C2C12 myoblasts, a cell model of fibrodysplasia ossificans progressiva. Bone 127, 592–601. doi: 10.1016/j.bone.2019.07.031 31376533

[ref18] Fátima A. Carlos R.-L. Rosario R. M. Vela A. I. Francisco F.-G. J. Leiva P. S.. (2018). Human *Pasteurella multocida* infection with likely zoonotic transmission from a pet dog, Spain. Emerg. Infect Dis. 24, 1145–1146. doi: 10.3201/eid2406.171998 29774848 PMC6004854

[ref19] García-Alvarez A. Vela A. I. San Martín E. Chaves F. Fernández-Garayzabal J. F. Lucas D.. (2017). Characterization of *Pasteurella multocida* associated with ovine pneumonia using multi-locus sequence typing (Mlst) and virulence-associated gene profile analysis and comparison with porcine isolates. Vet. Microbiol. 204, 180–187. doi: 10.1016/j.vetmic.2017.04.015 28532799

[ref20] Guo H. Wan H. Lou W. Khan R. U. You J. Huang B.. (2024). Deoxynivalenol and T-2 toxin cause liver damage and egg quality degradation through endoplasmic reticulum stress in summer laying hens. Int. J. Biometeorol. 68, 1387–1396. doi: 10.1007/s00484-024-02674-w, PMID: 38607562

[ref21] Hamilton T. D. Roe J. M. Hayes C. M. Webster A. J. (1998). Effects of ammonia inhalation and acetic acid pretreatment on colonization kinetics of toxigenic *Pasteurella multocida* within upper respiratory tracts of swine. J. Clin. Microbiol. 36, 1260–1265. doi: 10.1128/JCM.36.5.1260-1265.1998, PMID: 9574688 PMC104811

[ref22] Hannah H. Julia E. Gudula S. Klaus A. Joachim O. J. T. (2018). Involvement of osteocytes in the action of *Pasteurella multocida* toxin. Toxins (Basel) 10:328. doi: 10.3390/toxins10080328 30104531 PMC6115833

[ref23] Hennig B. Orth J. Aktories K. Diener M. (2008). Anion secretion evoked by *Pasteurella multocida* toxin across rat colon. Eur. J. Pharmacol. 583, 156–163. doi: 10.1016/j.ejphar.2008.01.017 18279849

[ref24] Hildebrand D. Heeg K. Kubatzky K. F. (2015). *Pasteurella multocida* toxin manipulates T cell differentiation. Front. Microbiol. 6:1273. doi: 10.3389/fmicb.2015.01273 26635744 PMC4652077

[ref25] Horiguchi Y. (2012). Swine atrophic rhinitis caused by *pasteurella multocida* toxin and bordetella dermonecrotic toxin. Curr. Top. Microbiol. Immunol. 361, 113–129. doi: 10.1007/82_2012_206, PMID: 22411430

[ref26] Hoskins I. C. Thomas L. H. Lax A. J. (1997). Nasal infection with *Pasteurella multocida* causes proliferation of bladder epithelium in gnotobiotic pigs. Vet. Rec. 140:22. doi: 10.1136/vr.140.1.22, PMID: 9004479

[ref27] Hsuan S. L. Liao C. M. Huang C. Winton J. R. Chen Z. W. Lee W. C.. (2009). Efficacy of a novel *Pasteurella multocida* vaccine against progressive atrophic rhinitis of swine. Vaccine 27, 2923–2929. doi: 10.1016/j.vaccine.2009.03.005, PMID: 19428902

[ref28] Kessoku T. Kobayashi T. Imajo K. Tanaka K. Yamamoto A. Takahashi K.. (2021). Endotoxins and non-alcoholic fatty liver disease. Front. Endocrinol. (Lausanne) 12:770986. doi: 10.3389/fendo.2021.770986, PMID: 34777261 PMC8586459

[ref29] Kitadokoro K. Kamitani S. Miyazawa M. Hanajima-Ozawa M. Fukui A. Miyake M.. (2007). Crystal structures reveal a thiol protease-like catalytic triad in the C-terminal region of *Pasteurella multocida* toxin. Proc. Natl. Acad. Sci. USA 104, 5139–5144. doi: 10.1073/pnas.0608197104, PMID: 17360394 PMC1829276

[ref30] Kubatzky K. F. (2022). *Pasteurella multocida* toxin - lessons learned from a mitogenic toxin. Front. Immunol. 13:1058905. doi: 10.3389/fimmu.2022.1058905, PMID: 36591313 PMC9800868

[ref31] Lariviere S. Leblanc L. Mittal K. R. Martineau G. P. (1992). Characterization of *Pasteurella multocida* from nasal cavities of piglets from farms with or without atrophic rhinitis. J. Clin. Microbiol. 30, 1398–1401. doi: 10.1128/jcm.30.6.1398-1401.1992 1624554 PMC265299

[ref32] Lax A. J. Grigoriadis A. E. (2001). *Pasteurella multocida* toxin: the mitogenic toxin that stimulates signalling cascades to regulate growth and differentiation. Int. J. Med. Microbiol. 291, 261–268. doi: 10.1078/1438-4221-00129 11680786

[ref33] Mbuthia P. G. Njagi L. W. Nyaga P. N. Bebora L. C. Minga U. Kamundia J.. (2008). *Pasteurella multocida* in scavenging family chickens and ducks: carrier status, age susceptibility and transmission between species. Avian Pathol. 37, 51–57. doi: 10.1080/03079450701784891 18202950

[ref34] Meng Z. Wang L. Liao Y. Peng Z. Li D. Zhou X.. (2021). The protective effect of Heme Oxygenase-1 on liver injury caused by Don-induced oxidative stress and cytotoxicity. Toxins (Basel) 13:732. doi: 10.3390/toxins13100732 34679025 PMC8541417

[ref35] Moustafa A. M. Torsten S. Simon G. Ben A. Marina H. Boyce J. D.. (2015). Comparative genomic analysis of Asian Haemorrhagic septicaemia-associated strains of *Pasteurella multocida* identifies more than 90 Haemorrhagic Septicaemia-specific genes. PLoS One 10:e0130296. doi: 10.1371/journal.pone.0130296 26151935 PMC4495038

[ref36] Orth J. H. C. Fester I. Siegert P. Weise M. Lanner U. Kamitani S.. (2013). Substrate specificity of *Pasteurella multocida* toxin for α subunits of heterotrimeric G proteins. FASEB J. 27, 832–842. doi: 10.1096/fj.12-213900 23150526 PMC3545528

[ref37] Osuna O. Edds G. T. Simpson C. F. (1982). Toxicology of aflatoxin B1, warfarin, and cadmium in young pigs: metal residues and pathology. Am. J. Vet. Res. 43, 1395–1400, PMID: 6808875

[ref38] Oubrahim H. Wong A. Wilson B. A. Chock P. B. (2012). mtorc1 plays a role in *Pasteurella multocida* toxin (Pmt)-induced protein synthesis and proliferation in Swiss 3T3 cells. J. Biol. Chem., 288, 2805–2815. doi: 10.1074/jbc.M112.427351 PMC3554945 23223576

[ref39] Peng Z. Liang W. Wang Y. Liu W. Zhang H. Yu T.. (2017). Experimental pathogenicity and complete genome characterization of a pig origin *Pasteurella multocida* serogroup F isolate Hn07. Vet. Microbiol. 198, 23–33. doi: 10.1016/j.vetmic.2016.11.028, PMID: 28062004

[ref40] Peng Z. Wang X. Zhou R. Chen H. Wilson B. A. Wu B. (2019). *Pasteurella multocida*: genotypes and genomics. Microbiol. Mol. Biol. Rev. 83:e00014-19. doi: 10.1128/MMBR.00014-19, PMID: 31484691 PMC6759666

[ref41] Pennings A. M. M. A. Storm P. K. J. V. M. (1984). A test in vero cell monolayers for toxin production by strains of *Pasteurella multocida* isolated from pigs suspected of having atrophic rhinitis. Vet. Microbiol. 9, 503–508. doi: 10.1016/0378-1135(84)90071-3 6495611

[ref42] Preu I. Hildebrand D. Orth J. H. C. Aktories K. Kubatzky K. F. J. C. M. (2010). *Pasteurella multocida* toxin is a potent activator of anti-apoptotic signalling pathways. Cell Microbiol 12, 1174–1185. doi: 10.1111/j.1462-5822.2010.01462.x 20331638

[ref43] Rajeev S. Nair R. V. Kania S. A. Bemis D. A. (2003). Expression of a truncated *Pasteurella multocida* toxin antigen in *Bordetella bronchiseptica*. Vet. Microbiol. 94, 313–323. doi: 10.1016/S0378-1135(03)00137-8, PMID: 12829385

[ref44] Repella T. L. Ho M. Chong T. P. M. Bannai Y. Wilson B. A. J. T. (2011). Arf6-dependent intracellular trafficking of *Pasteurella multocida* toxin and pH-dependent translocation from late endosomes. Toxins (Basel) 3, 218–241. doi: 10.3390/toxins3030218 22053287 PMC3202820

[ref45] Rutter J. M. (1983). Virulence of *Pasteurella multocida* in atrophic rhinitis of gnotobiotic pigs infected with *Bordetella bronchiseptica*. Res. Vet. Sci. 34:287. doi: 10.1080/00480169.1983.35041. 6878879

[ref46] Rutter J. M. Mackenzie A. (1984). Pathogenesis of atrophic rhinitis in pigs: a new perspective. Vet. Rec. 114, 89–90. doi: 10.1136/vr.114.4.89, PMID: 6719815

[ref47] Sakano T. Okada M. Taneda A. Ono M. Sato S. (1992). Experimental atrophic rhinitis in 2 and 4 month old pigs infected sequentially with Bordetella bronchiseptica and toxigenic type D *Pasteurella multocida*. Vet. Microbiol. 31, 197–206. doi: 10.1016/0378-1135(92)90078-8, PMID: 1385667

[ref48] Schoellkopf J. Mueller T. Hippchen L. Mueller T. Reuten R. Backofen R.. (2022). Genome wide Crispr screen for *Pasteurella multocida* toxin (Pmt) binding proteins reveals Ldl receptor related protein 1 (Lrp1) as crucial cellular receptor. PLoS Pathog. 18:e1010781. doi: 10.1371/journal.ppat.1010781, PMID: 36516199 PMC9797058

[ref49] Seo B. Choy E. W. Maudsley S. Miller W. E. Wilson B. A. Luttrell L. M. (2000). *Pasteurella multocida* toxin stimulates mitogen-activated protein kinase via Gq/11-dependent transactivation of the epidermal growth factor receptor. J. Biol. Chem. 275, 2239–2245. doi: 10.1074/jbc.275.3.2239 10636931

[ref50] Siegert P. Schmidt G. Papatheodorou P. Wieland T. Aktories K. Orth J. H. C.. (2013). *Pasteurella Multocida* toxin prevents osteoblast differentiation by transactivation of the map-kinase Cascade via the Gαq/11 - p63Rhogef - RhoA Axis. PLoS Pathog. 9:e1003385. doi: 10.1371/journal.ppat.1003385 23696743 PMC3656108

[ref51] Strack J. Heni H. Gilsbach R. Hein L. Orth J. H. C. J. M. (2014). Noncanonical G-protein-dependent modulation of osteoclast differentiation and bone resorption mediated by *Pasteurella multocida* toxin. mBio 5:e02190-14-e02190-14. doi: 10.1128/mBio.02190-14 25389180 PMC4235216

[ref52] Surguy S. M. Duricki D. A. Reilly J. M. Lax A. J. Robbins J. J. N. (2014). The actions of *Pasteurella multocida* toxin on neuronal cells. Neuropharmacology 77, 9–18. doi: 10.1016/j.neuropharm.2013.09.005 24055502 PMC3878393

[ref53] Sushmita C. Bianca K. Ulrike H. Georg S. Kubatzky K. F. (2017). *Pasteurella multocida* Toxin Triggers Rankl-Independent Osteoclastogenesis. Front. Immunol. 8:8:185. doi: 10.3389/fimmu.2017.00185 28289415 PMC5327351

[ref54] Thomson J. R. Macintyre N. Henderson L. E. Meikle C. S. (2001). Detection of *Pasteurella multocida* in pigs with porcine dermatitis and nephropathy syndrome. Vet. Rec. 149, 412–417. doi: 10.1136/vr.149.14.412 11678213

[ref55] To H. Someno S. Nagai S. (2005). Development of a genetically modified nontoxigenic *Pasteurella multocida* toxin as a candidate for use in vaccines against progressive atrophic rhinitis in pigs. Am. J. Vet. Res. 66, 113–118. doi: 10.2460/ajvr.2005.66.113 15691045

[ref56] Truswell A. Laird T. J. Jones S. O'dea M. Blinco J. Abraham R.. (2023). Antimicrobial resistance of and genomic insights into *Pasteurella multocida* strains isolated from Australian pigs. Microbiol. Spectr. 11:e0378422. doi: 10.1128/spectrum.03784-22, PMID: 36651773 PMC9927299

[ref57] Wang F. Zhao S. Deng D. Wang W. Xu X. Liu X.. (2021). Integrating lcm-based Spatio-temporal Transcriptomics uncovers conceptus and endometrial luminal epithelium communication that coordinates the conceptus attachment in pigs. Int. J. Mol. Sci. 22:1248. doi: 10.3390/ijms22031248 33513863 PMC7866100

[ref58] Wang Y. Zhou C. Fu Y. Zhang L. Liu S. Cai L.. (2023). Establishment of acute liver failure model in Tibetan miniature pig and verified by dual plasma molecular adsorption system. Int. J. Artif. Organs 46, 141–152. doi: 10.1177/03913988221145501, PMID: 36600401

[ref59] Weise M. Vettel C. Spiger K. Gilsbach R. Hein L. Lorenz K.. (2015). A systemic *Pasteurella multocida* toxin aggravates cardiac hypertrophy and fibrosis in mice. Cell. Microbiol. 17, 1320–1331. doi: 10.1111/cmi.12436, PMID: 25759205

[ref60] Wilkie I. W. Harper M. Boyce J. D. Adler B. (2012). *Pasteurella multocida*: Diseases and pathogenesis. Curr. Top. Microbiol. Immunol. 361, 1–22. doi: 10.1007/82_2012_216 22643916

[ref61] Williams P. P. Hall M. R. Rimler R. B. (1990). Host response to *Pasteurella multocida* turbinate atrophy toxin in swine. Can. J. Vet. Res. 54, 157–163, PMID: 2306667 PMC1255621

[ref62] Wilson B. A. Ho M. (2012). *Pasteurella multocida* toxin interaction with host cells: entry and cellular effects. Curr. Top. Microbiol. Immunol. 361, 93–111. doi: 10.1007/82_2012_219, PMID: 22552700 PMC4408768

[ref63] Wilson B. A. Ho M. (2013). *Pasteurella multocida*: from zoonosis to cellular microbiology. Clin. Microbiol. Rev. 26, 631–655. doi: 10.1128/CMR.00024-13 23824375 PMC3719492

[ref64] Xiao H. Zhao Q. Yuan J. Liang W. Wu R. Wen Y.. (2023). Ifn-γ promotes Panoptosis in *Pasteurella multocida* toxin-induced pneumonia in mice. Vet. Microbiol. 285:109848. doi: 10.1016/j.vetmic.2023.109848, PMID: 37722207

[ref65] Yuan J. Zhao Q. Li J. Wen Y. Wu R. Zhao S.. (2024). Cxcl8 knockout: a key to resisting *Pasteurella multocida* toxin-induced cytotoxicity. Int. J. Mol. Sci. 25:5330. doi: 10.3390/ijms25105330, PMID: 38791369 PMC11121343

[ref66] Zhu Q. Ma Y. Liang J. Wei Z. Li M. Zhang Y.. (2021). Ahr mediates the aflatoxin B1 toxicity associated with hepatocellular carcinoma. Signal Transduct. Target. Ther. 6:299. doi: 10.1038/s41392-021-00713-1, PMID: 34373448 PMC8352983

