# Supplementary material for: Revealing the lethal effects of Pasteurella multocida toxin on multiple organ systems
Source: Front Microbiol. 2024 Aug 27;15:1459124. doi: 10.3389/fmicb.2024.1459124 (PMC11385013; doi:10.3389/fmicb.2024.1459124)
Supplement: Supplementary file 1 [file Data_Sheet_1.PDF]

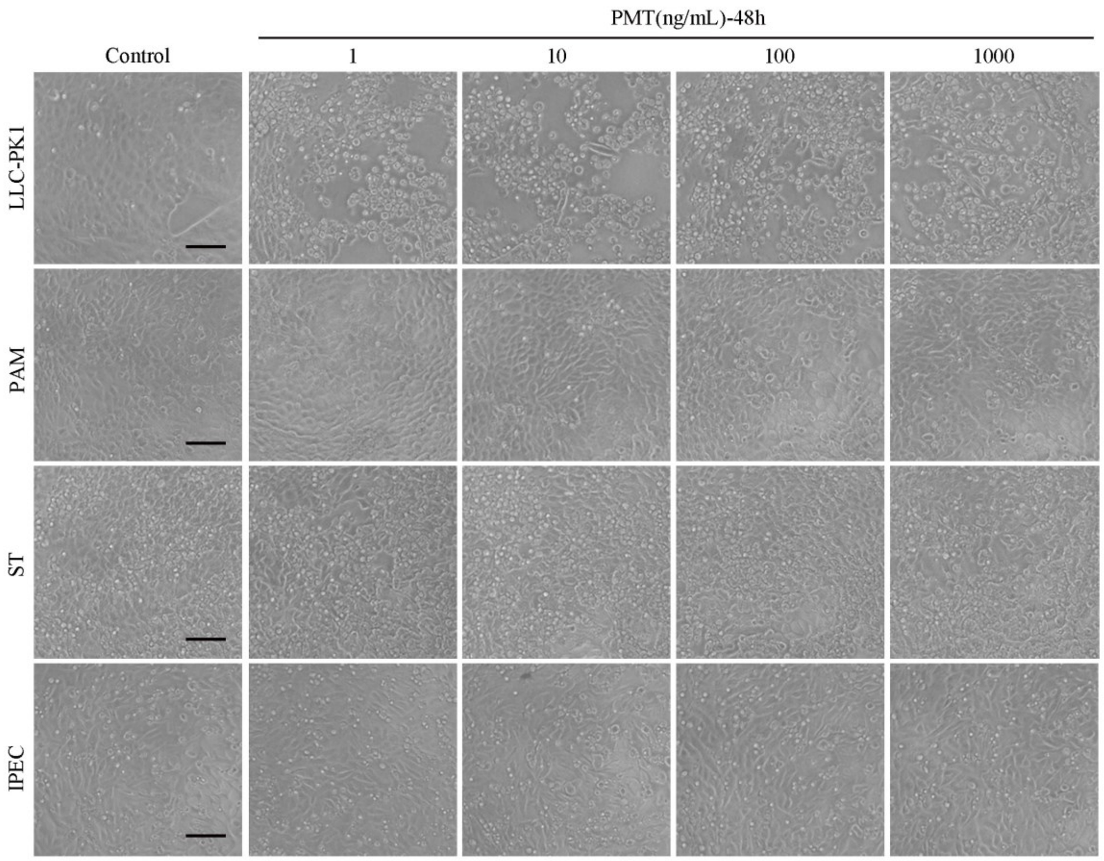

Figure S1. PMT cytotoxicity on LLC-PK1, PAM, ST and IPEC cells

LLC-PK1, PAM, ST and IPEC Cells were incubated with 1, 10, 100 and 1000 ng/mL of PMT (48 h; 37 °C) and observed under a microscope. Scale bar, 100 μm.

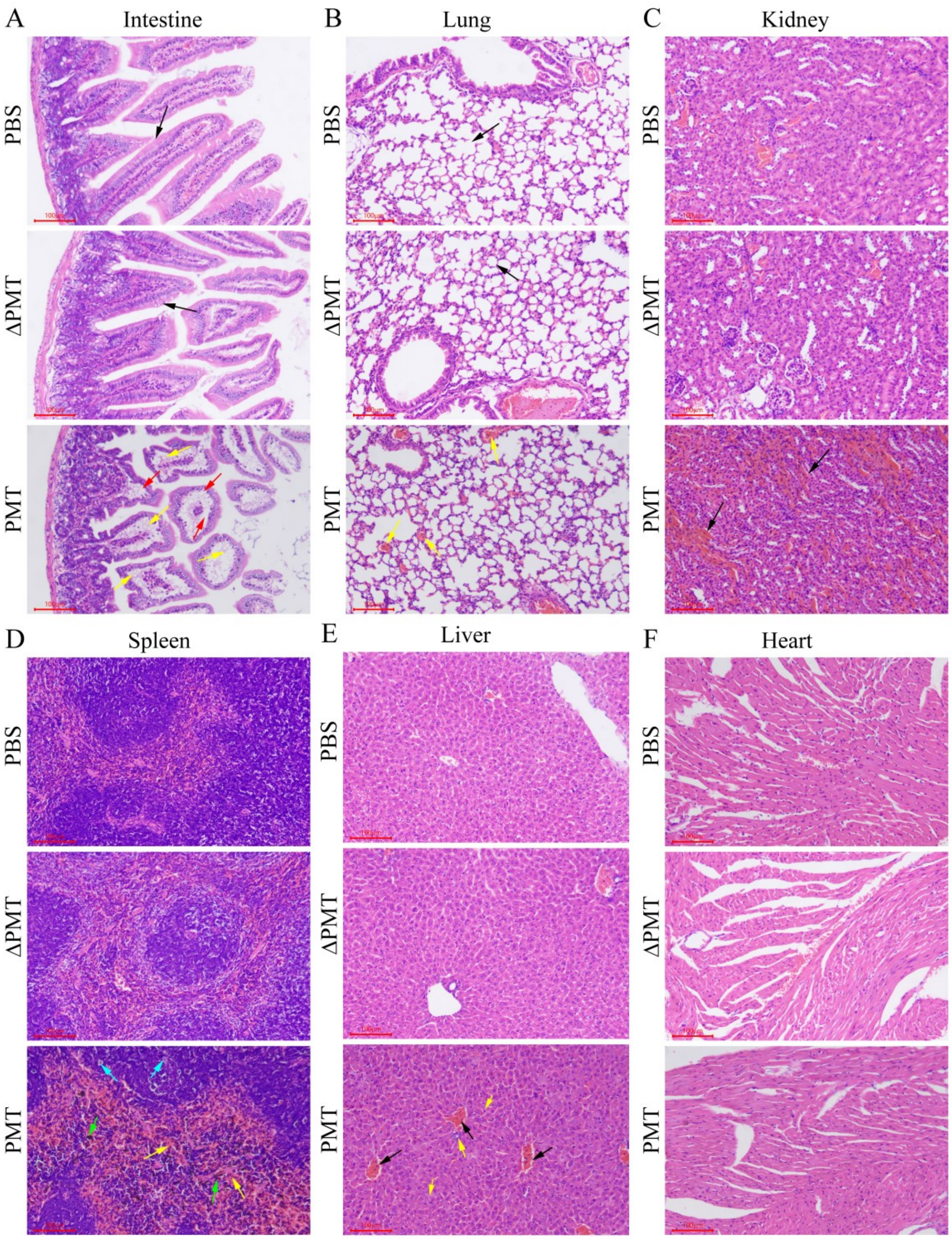

Figure S2. Pathological damage to mouse organs after treatment with PMT. Hematoxylin and eosin (H&E) was used to stain the intestines, lungs, kidneys, spleens, livers and hearts of the mice after i.p. injection with 0.5 μg/kg PMT (body weight), 0.5 μg/kg ΔPMT (body weight) or PBS. (A) The normal and damage of intestines tissue was marked with different color arrows. (B) The normal and damage of lung tissue was marked with different color arrows. (C) The damage of kidney tissue was marked with

black arrows. (D) The damage of spleen tissue was marked with different color arrows. (E) The damage of liver tissue was marked with black and yellow arrows. (F) The heart tissue with PMT treated and untreated. Scale bar, 100 μm.

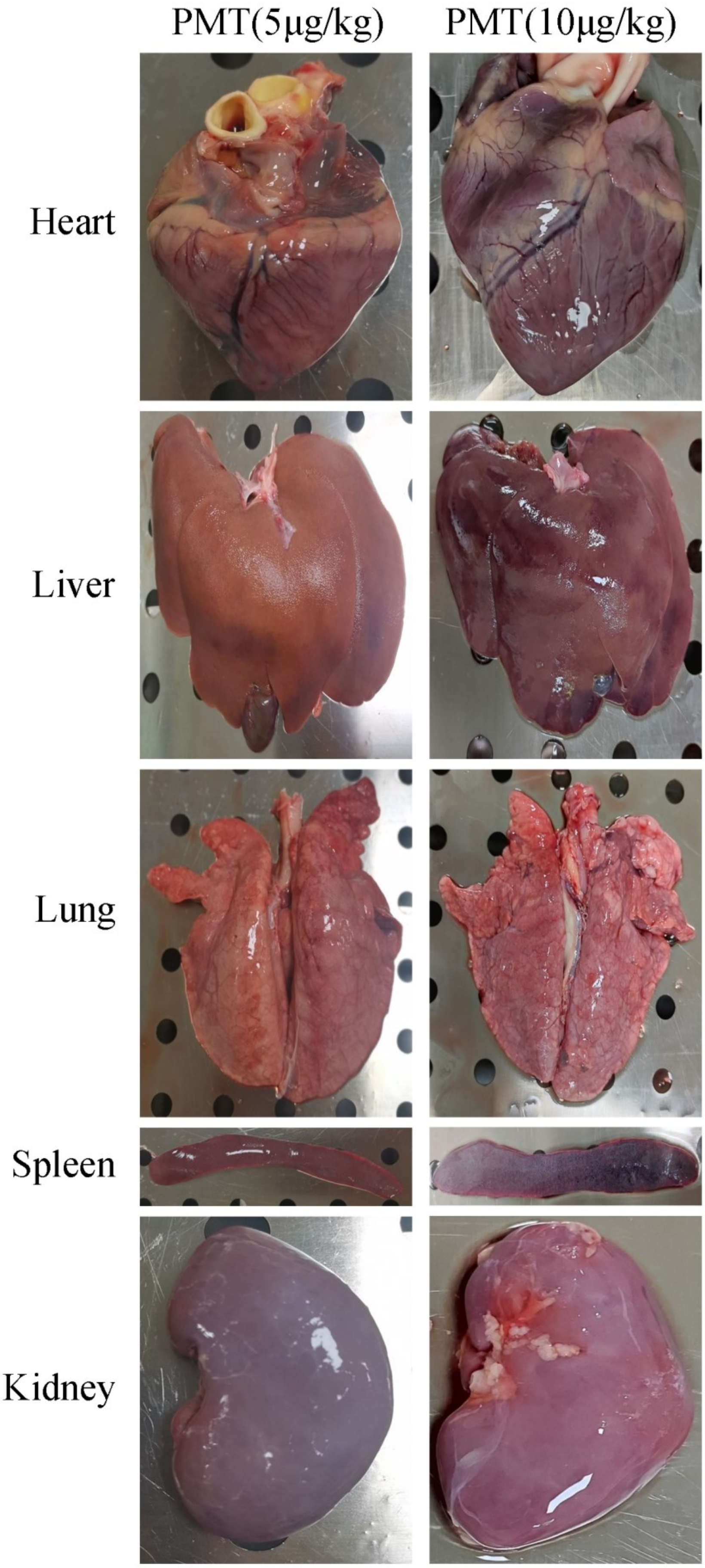
PMT(5μg/kg)
PMT(10μg/kg)
Heart
Liver
Lung
Spleen
Kidney

Figure S3. Toxic effects of PMT on piglet organs. The piglets were administered different concentrations of PMT. The piglets exposed to 5 μg/kg and 10 μg/kg (body weight) PMT were promptly dissected after death. Organs, including the heart, liver, spleen, lung, and kidney, were extracted for photographic documentation.

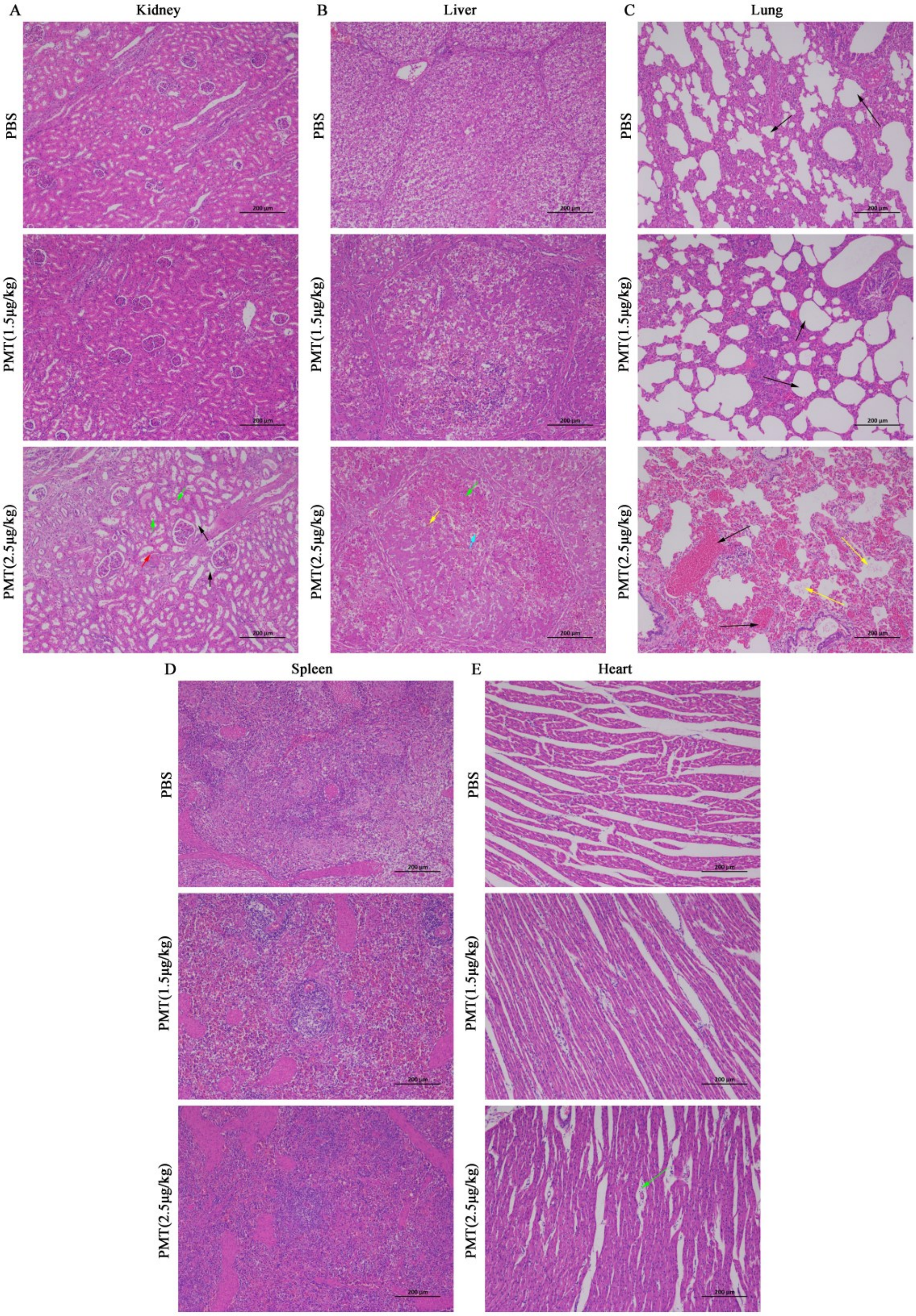

Figure S4. HE pathological staining of organs and tissues in pigs. Hematoxylin and eosin (H&E) were used to stain the lungs, kidneys, spleens, livers and hearts of piglets after injection with PMT at 1.5 μg/kg or 2.5 μg/kg (body weight) or PBS after 14 days. (A) The normal and damage of kidney tissue was marked with different color arrows. (B) The damage of liver tissue was marked with different color arrows. (C) The normal and damage of lung tissue was marked with different color arrows. (D) The spleen tissue with PMT treated and untreated. (E) The damage of heart tissue was marked with green arrows. Scale bar, 200 μm.
